# A *Sorghum bicolor* expression atlas reveals dynamic genotype-specific expression profiles for vegetative tissues of grain, sweet and bioenergy sorghums

**DOI:** 10.1186/1471-2229-14-35

**Published:** 2014-01-23

**Authors:** Nadia Shakoor, Ramesh Nair, Oswald Crasta, Geoffrey Morris, Alex Feltus, Stephen Kresovich

**Affiliations:** 1 Chromatin Inc, Chicago, Illinois, USA; 2 Department of Biological Sciences, The University of South Carolina, 715 Sumter Street, Columbia, SC 29208, USA; 3 Current address: Dow AgroSciences, Indianapolis, Indiana, USA; 4 Department of Genetics and Biochemistry, Clemson University, Clemson, South Carolina, USA

**Keywords:** *Sorghum bicolor*, Gene atlas, Transcriptome, Gene expression, Functional genomics, Microarray

## Abstract

**Background:**

Effective improvement in sorghum crop development necessitates a genomics-based approach to identify functional genes and QTLs. Sequenced in 2009, a comprehensive annotation of the sorghum genome and the development of functional genomics resources is key to enable the discovery and deployment of regulatory and metabolic genes and gene networks for crop improvement.

**Results:**

This study utilizes the first commercially available whole-transcriptome sorghum microarray (Sorgh-WTa520972F) to identify tissue and genotype-specific expression patterns for all identified *Sorghum bicolor* exons and UTRs. The genechip contains 1,026,373 probes covering 149,182 exons (27,577 genes) across the *Sorghum bicolor* nuclear, chloroplast, and mitochondrial genomes. Specific probesets were also included for putative non-coding RNAs that may play a role in gene regulation (*e.g.,* microRNAs), and confirmed functional small RNAs in related species (maize and sugarcane) were also included in our array design. We generated expression data for 78 samples with a combination of four different tissue types (shoot, root, leaf and stem), two dissected stem tissues (pith and rind) and six diverse genotypes, which included 6 public sorghum lines (R159, Atlas, Fremont, PI152611, AR2400 and PI455230) representing grain, sweet, forage, and high biomass ideotypes.

**Conclusions:**

Here we present a summary of the microarray dataset, including analysis of tissue-specific gene expression profiles and associated expression profiles of relevant metabolic pathways. With an aim to enable identification and functional characterization of genes in sorghum, this expression atlas presents a new and valuable resource to the research community.

## Background

Sorghum [S*orghum bicolor* (L.) Moench] is a staple cereal crop for millions of people in the marginal, semi-arid environments of Africa and South Asia. Its unique and advanced ability to grow in regions of low and variable rainfall highlight its potential to impact agricultural productivity in widespread water-limited environments [1,2]. Originating and evolving across the diverse environmental landscape of Africa, morphological and physiological adaptation strategies has advanced sorghum as a naturally heat and drought-tolerant warm season C_4_ grass that is more efficient at utilizing water, nitrogen and energy resources with respect to other major crops, including maize and wheat [1,3,4]. Occupying seven million hectares of farmland, the United States is currently the world’s top sorghum producer (8.8 million annual metric tons), followed by India (7.0), Mexico (6.9), and Nigeria (4.8) (http://cgiar.org/sorghum). Cultivated in diverse climates and environmental conditions, the challenges of increasing performance and yield on marginal lands and cooler climates remains at the forefront of sorghum improvement efforts worldwide [5,6].

Sorghum is globally established as an important source of food, feed, sugar and fiber, and recent interest in bioenergy feedstocks also spotlights sorghum as an attractive alternative for sustainable biofuel production [4]. Framed upon the 2009 sorghum reference genome [7], translational genomic resources have been developed that directly impact research in other closely related C_4_ feedstock grasses, including switchgrass and *Miscanthus*[8,9]. Comprehensive understanding of the genetic and molecular mechanisms that regulate metabolite biosynthesis, transport and storage in these species is essential for the efficient development of biofuel feedstocks.

Global transcriptome profiling further provides a means to access gene networks for the discovery of functional connections between genes, mRNAs and their regulatory proteins, and complex traits expressed through coordinated and dynamic gene networks across different tissues and developmental stages [10]. Over the last decade, microarray-based expression profiling has provided a reliable high-throughput platform for genome-wide analysis of gene expression in many organisms. Microarrays offer substantial advantages for functional genomics, as they are increasingly cost-effective, provide a comparable accuracy of expression profiling to RNA-sequencing, and have been shown to provide comprehensive expression data (up to 90% of the transcriptome) in a given tissue [11]. Well-established microarray data analysis tools are also available for querying, visualizing and analyzing the genomes and predicted genes [12,13], as well as for analyzing the transcriptome profiling data and integrating with other public datasets [14-17].

To provide insight into the sorghum transcriptome, we generated a record of gene expression in a set of seven tissues and six diverse sorghum genotypes. The choice of samples reflects our aim to develop and enrich the current sorghum transcriptome literature. Previous studies have predominantly focused on reproductive tissues, and the majority of these reports do not represent the complete sorghum transcriptome. Several of these studies have also been limited to the reference genome (BTx623) or Keller, a recently resequenced sweet sorghum variety [18-22].

Comparable whole plant transcriptome maps are available for a number of other model species, including *Arabidopsis thaliana*[23], maize (*Zea mays)*[24], barley (*Hordeum vulgare*) [25], rice (*Oryza sativa*) [26,27], and soybean (*Glycine max*) [28]. These recent transcriptome surveys were constructed with only one genotype or line/accession for their respective species of interest, whereas the present study aims to highlight the practical importance of examining expression profiles across diverse tissue types, developmental stages, as well as genotypes in order to accurately target genes and metabolic pathways for the efficient development of improved feedstocks.

Fundamental understanding of sorghum genomics is necessary for improving sorghum for agronomic and compositional traits. Specifically, genotypes with high biomass and increased levels of fermentable stem sugars are ideal for developing feedstocks for the biofuel industry. We developed this genomic resource, the whole-transcriptome array as well as the vegetative transcriptome in diverse genotypes and tissues, in order to facilitate the characterization of molecular networks and regulatory mechanisms governing important metabolic pathways including, but not limited to, cell wall biosynthesis for lignocellulosic biomass as well as synthesis, translocation, and storage of fermentable photosynthates for energy content. The relevance of our dataset is demonstrated by genotype and tissue-specific expression of the phenylpropanoid and lignin biosynthetic pathway genes.

Intended as readily available public resource for functional gene characterization, the transcriptome data presented here is available through NCBI's Gene Expression Omnibus (GEO) under accession number GSE49879, and the Sorghum Genome Array is available through Affymetrix (http://affymetrix.com).

## Results and discussion

### Generation and quality assessment of data

A whole-transcriptome exon array for *Sorghum bicolor* was custom-designed by Chromatin, Inc. (http://chromatininc.com) and Affymetrix: *Sorgh-WTa520972F*. This genechip contains 1,026,373 probes covering 149,182 exons (27,577 genes) across the *Sorghum bicolor* genome (10 chromosomes), chloroplast and mitochondria. The sequences used to construct the probesets included all identified *Sorghum bicolor* exons from the Sbi1 assembly (http://www.phytozome.net). Multiple probes were chosen for each exon, with a minimum of one probe per exon and 25 probes per gene. In addition to standard Affymetrix controls, positive controls in the microarray design included probes for constitutively expressed *Sorghum bicolor* genes (actin, ubiquitin and eIF4a1). Probes for intronic regions of actin and ubiquitin were also utilized to determine background expression levels.

To study the sorghum transcriptome and build a gene expression atlas, we collected 78 diverse samples from various developmental stages and tissue types (Additional file 1). In order to broadly capture sorghum genetic diversity, we included genotypes representing three major ideotypes, including grain, sweet, and bioenergy sorghums. Our study includes R159, an elite grain sorghum characterized by the valuable agronomic traits of uniform growth and disease resistance [29]. Grain sorghum is cultivated primarily for its high starch content, applications in human/animal health and nutrition, and as biofuel feedstock for ethanol production [5]. We also included two sweet sorghums, Fremont and Atlas, that produce increased biomass and accumulate high levels of fermentable carbohydrates in the stem. Additionally, Fremont is drought resistant and flowers early, while Atlas is less susceptible to lodging (due to a stiff stalk phenotype) and flowers later [30]. We also selected three bioenergy or high biomass lines, PI455230, PI152611, and AR2400 that produce increased levels of cellulosic material and are photoperiod sensitive, which allows the plant to produce higher amounts of vegetative matter under long day conditions (Additional file 2). PI152611 is specifically a forage line, a fast-growing, highly digestible grass utilized for livestock feed [5,29].

The primary goal of this study was to obtain relevant and applicable data for the research community developing sorghum as a global feedstock; this research interest guided our sample selection towards vegetative tissues, with a strong bias for stem tissues. A comprehensive trancriptomic profile of sorghum inflorescence and leaf data was recently made available to the community [19]. We compared the leaf RNA sequencing dataset with the present leaf dataset to demonstrate and confirm that our microarray analysis approach towards transcriptome profiling was appropriate. The Spearman correlation of the transcriptome across technologies is 0.61 (Additional file 3), which is consistent with several studies comparing RNA-seq and microarray methods for genome-wide transcriptome profiling [31-33]. The present comparison corroborates these studies and demonstrates that the microarray platform for expression profiling correlates well with current sequencing methods. With a common goal of crop improvement, complementary datasets such as these generate a core of information that can be explored for the functional characterization of genes and genetic pathways.

We assessed data quality for hybridization by comparing normalized signals of all probe sets between biological replicates using Pearson’s correlation analysis. The biological replicates were highly correlated, with an average Pearson’s correlation coefficient of 0.99 (Additional file 4). The highly reproducible results of the replicate data further validate the quality of the microarray platform and present dataset. Previous studies have consistently established strong correlations between qRT-PCR data and microarray data processed using robust multi-array analysis (RMA) [34,35]. However, we also tested a small subset of these genes via qRT-PCR to validate the array-generated expression data and expression patterns across multiple tissue types (Additional file 5A and 5B).

To further assure data quality, we also examined the general expression patterns of well-characterized genes that have been highlighted for tissue-specific expression in previous studies. In microarray experiments with RNA isolated from shoot tips, we observed high expression levels for homologs of SPATULA, a shoot tip transcription factor that is strongly expressed in shoot tips and young leaf primordia [36]. Similarly, the sorghum homolog for TIP2-3, a root-specific aquaporin gene [37], was also expressed at higher levels in our study using root-isolated RNA (Additional file 6).

### Global gene expression patterns

We detected the expression of 19,354 genes in at least one of the 78 samples, representing 70.2% of all genes on the array (27,577 genes). The number of expressed transcripts detected in the various tissues ranged between 10,850 and 11,587 (representing 56 to 60% of all expressed genes on the array). Expressed genes were determined following established methods [24], and with a conservative and arbitrary expression threshold cutoff of 320 (five times the mean normalized signal from intronic gene probes used as controls), we found that 15.4% of genes on the array were detected in all tissues (4256/27,577) (Additional file 7). Gene ontology (GO) annotation analysis of these constitutively expressed genes reveals that most are involved in basic biological processes including development, protein synthesis/modification, and signal transduction (Additional file 8). Similar to published work in maize, expression of constitutive genes varied among the samples, with the coefficient of variation (CV) ranging from 5% to 129%. With a CV of 10.4%, we identified a ubiquitin-conjugating enzyme, Sb09g023560, as one of the most stably expressed genes (Additional file 9). This class of genes was also identified in the maize atlas as the most stably expressed among variable tissues [24].

A diverse range of plant tissues was sampled in this study; however, 29.8% of the probesets were not detected above our designated expression threshold level. Several plausible explanations can account for this incomplete expression coverage, including gene expression from specific tissues and/or developmental stages not included in this study, false positive gene models, and levels of expression below detection threshold limits. Further, the arrays were developed utilizing the BTx623 reference sequence and do not capture polymorphisms, copy number variation and presence-absence variation across all the sampled genotypes.

### Transcriptome-based classification of sorghum tissues

**Figure 1:**
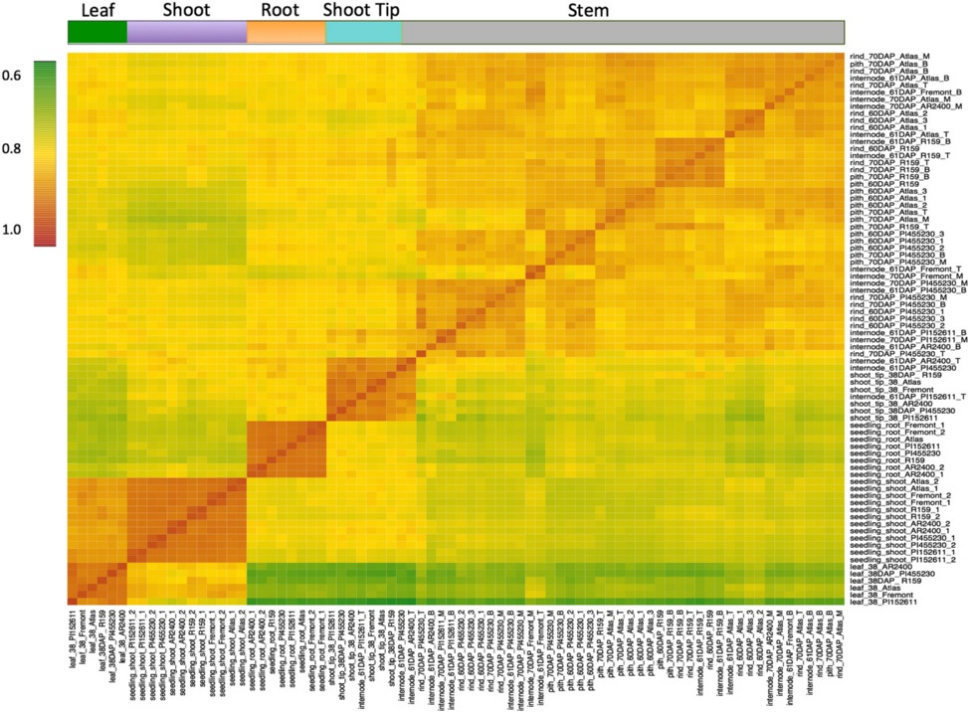
**Pearson’s correlation matrix of the whole dataset.** Pair-wise Pearson correlation coefficients were calculated from the gene expression values of the whole transcriptome (27,577 genes) in all 78 samples. The hierarchical clusters were obtained based on Euclidian distance and are indicated by the color bar on the top side of the figure. The color scale indicates the degree of correlation.

**Figure 2:**
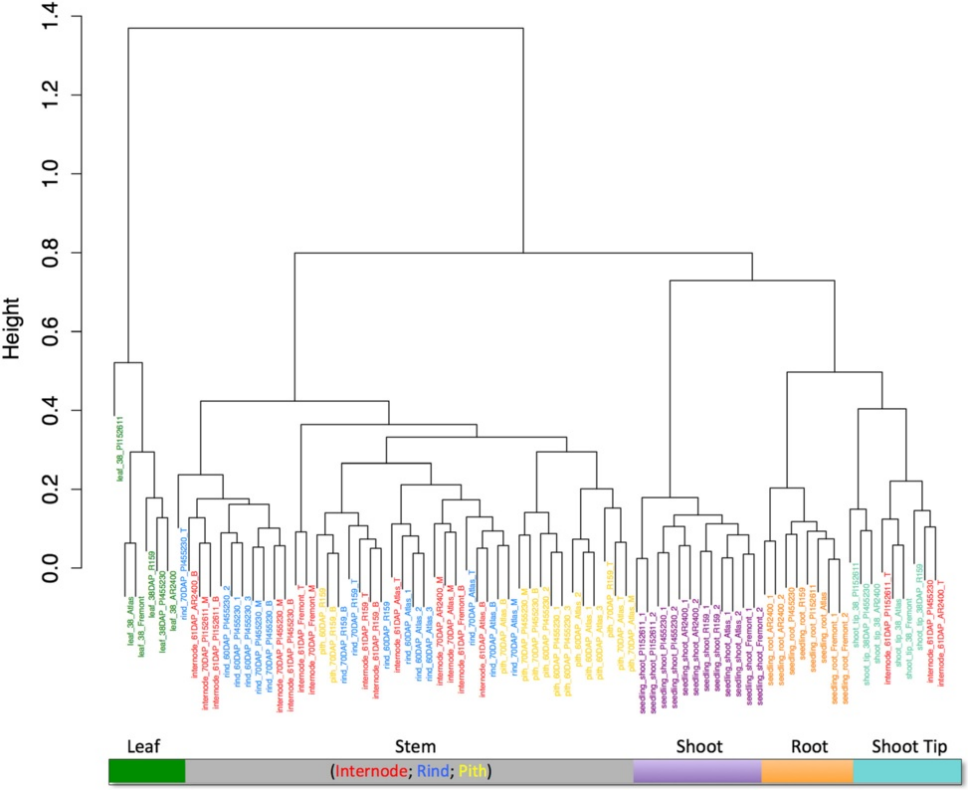
**Cluster dendrogram of the whole dataset (78 samples).** The hierarchical clusters of organs were grouped based on Euclidian distance. The 5 clusters are indicated by the color bar on the bottom side of the figure.

**Figure 3:**
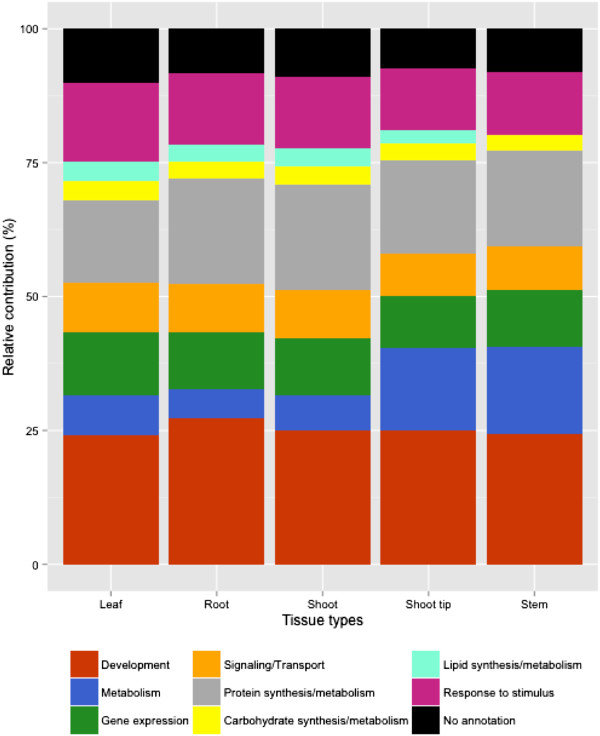
**Functional category distribution of tissue-specific transcripts.** Expression levels of select Gene Ontology categories across tissue types. The Sbi1.4 version of the sorghum annotation allowed for the identification of ~85% of expressed genes across all tissue types. The transcripts were manually verified and grouped into 7 functional categories based on Plant GO slim classifications.

A Pearson’s distance correlation matrix was constructed to compare and evaluate the transcriptome data from each sample (Figure 1). This data shows strong correlations among and within the individual tissue types. The associated dendrogram reveals clustering according to tissue type as well as genotype, highlighting the significance of genotype-specific expression in this study (Figure 2). Utilizing GO categories, functional analysis of the identified gene sets revealed enrichment of known tissue-specific biological processes. For example, the leaf and shoot-associated gene sets were enriched for photosynthetic genes relative to the roots, as expected (Additional file 8). We found that components of protein synthesis were overexpressed in the seedling roots and shoots, whereas genes involved in metabolism were over-represented in the shoot tip and stem tissues (Figure 3). These data identify core sets of genes associated with various biological processes and are clear targets for future study aimed to definitively characterize their functions in specific tissues.

**Figure 4:**
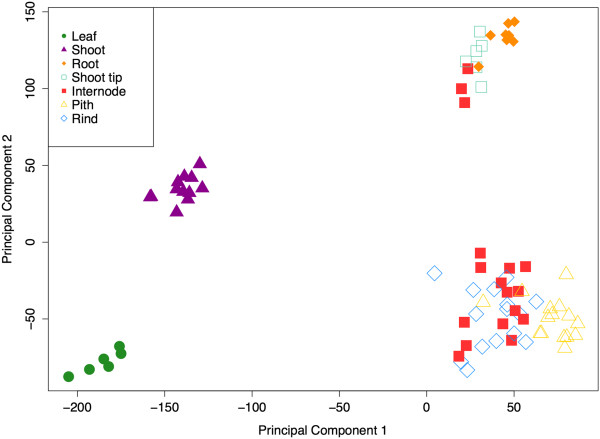
**Classes sharing similar expression patterns.** Principal component analysis was applied to 78 tissue samples, based on expression of 29,065 probe sets (27,577 genes, 654 controls and 834 small RNA probe sets). Each symbol represents a single sample. Tissue types are indicated by color and shape of symbol.

Differential transcriptomes of developmentally distinct vegetative tissues were also apparent from the principal component analysis (PCA) (Figure 4). The PCA reveals clustering of functionally related tissue types, and the first two principal components (PC) of this analysis explain 68% of the variance among samples (PC1 = 48%, PC2 = 20%). Apical meristematic zones of the roots and shoot tips clustered together and weakly clustered with leaves, shoots and stem tissues. The large group of stem tissues (46 samples) including internode, pith, and rind strongly clustered together and weakly with the remaining tissues. These results are consistent with previous studies in maize and *P. halli* crop models, that show core similarities among stem-associated tissues and subsequent divergence of root and leaf samples [24,38].

Interestingly, three out of 46 stem samples clustered near the group of meristematic tissues (roots and shoot tips). All three of these 'outlier’ samples were collected at the top internode, 61 days after planting (DAP) in three of the six sampled genotypes (PI455230, PI152611, and AR2400). At 70DAP, the stem samples from same genotypes clustered with the other stem samples. These lines are characterized as high biomass genotypes, whereas the remaining three genotypes can be characterized as either grain or sweet lines (R159, Atlas, and Fremont). The PCA indicates that at 61DAP, the patterns of gene expression in the stem of the high biomass lines are more related to meristematic regions, or regions of active growth. While it is possible that these three stem samples were collected too close to the meristematic shoot tip region, further study may indicate that the differential transcriptome in the stems of these lines capture a transition zone of gene expression in which sorghum commits to post-reproductive pathways of sugar production and grain fill versus continued biomass production. This result further demonstrates the importance of examining genotype, tissue type, as well as temporal expression patterns when targeting transcriptional programs of interest.

### Tissue and genotype-specific patterns of gene expression

**Figure 5:**
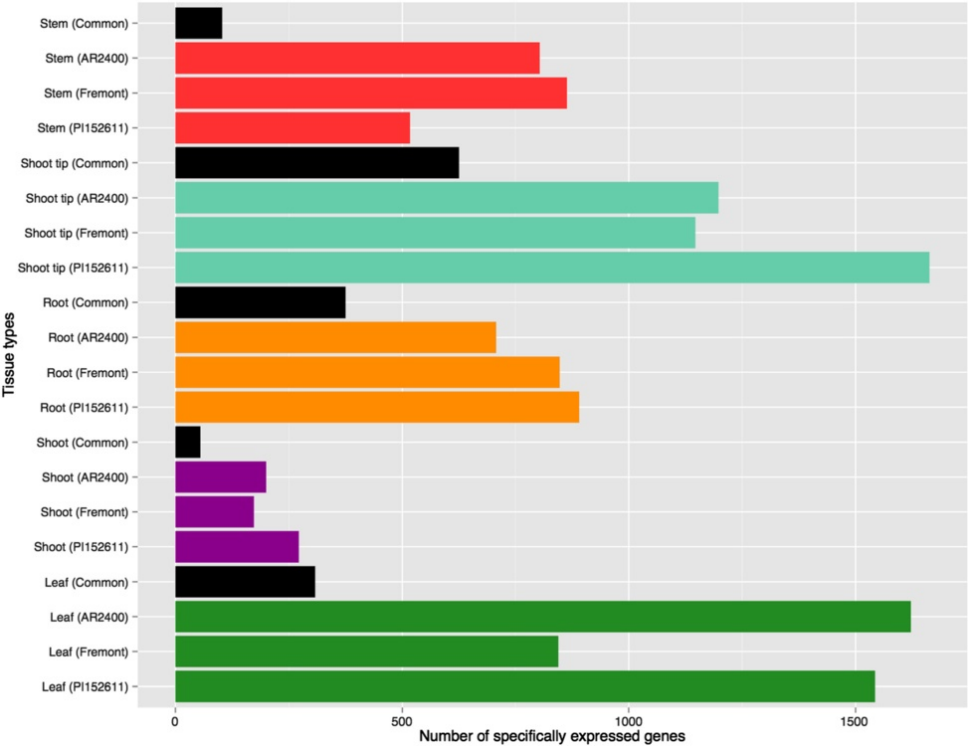
**Number of tissue-specific genes in across sorghum ideotypes.** AR2400: biomass sorghum; Fremont: sweet sorghum; PI152611: forage sorghum; Common: number of genes in common among all three ideotypes.

To identify tissue-specific genes, we created genotype-specific datasets for PI152611, Fremont, and AR2400, each representing one of three major classes of sorghum: forage, sweet, and high biomass types respectively. Excluding replicate tissues from the same major organ, we identified genes exclusively expressed in the leaf, shoot, root, shoot tip and stem (Figure 5). The leaf and meristematic shoot tips expressed the greatest number of tissue-specific transcripts across all three genotypes, whereas the seedling shoots expressed the fewest number of tissue-specific genes. Of particular interest in this dataset is the extent of variation observed across genotypes. For example, in stems, over 800 stem-specific genes are identified in representative examples of sweet and high biomass sorghum. Over 500 stem-specific genes are detected in forage sorghum; however, only 103 stem-specific genes are common among all three sorghum types. This lack of shared tissue-specific genes across genotypes is observed in all major tissue types. We also carried out this analysis for the small RNAs included on the array (Additional file 10). Similar to gene expression, we observed both tissue and genotype-specific expression of the small RNAs (Additional file 11). For purposes of functional crop improvement, these results highlight the significance of intra-species variation in sorghum and the importance of selecting the appropriate genotype for targeted changes to gene expression via transgenic and breeding approaches.

**Figure 6:**
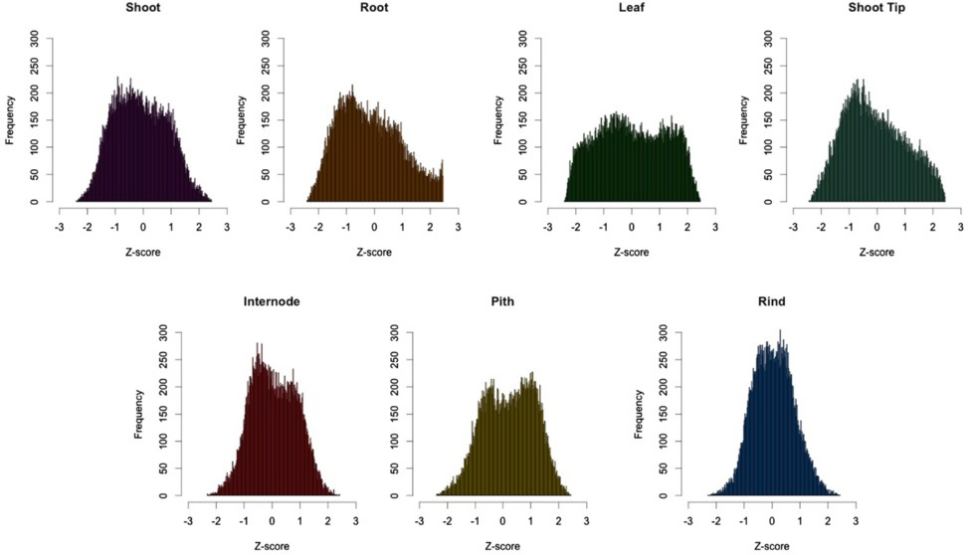
**Distribution of global gene expression across sorghum tissue types.** Histograms of relative expression levels (measured by Z-scores) in each tissue type. For each of these tissues, Z scores were calculated as follows: Z = (X-X_mean_)/SD, where X is the average expression of a given gene in a tissue, and X_mean_ and SD are the mean expression and standard deviation respectively of that gene across all the selected tissues.

To illustrate the expression dynamics among tissues, we also calculated the relative gene expression levels (Z-scores) of each of the major tissues (Figure 6). Consistent with previous studies, tissues with a relatively higher number of tissue-specific genes (e.g. leaf, root, shoot tip, pith) had a wide distribution of genes deviating from their mean expression. Stem-associated tissues had similar expression profiles and gene expression was closer to the overall average across tissue types [24,38].

We next attempted to determine whether functional gene classes were over-represented in specific genotypes. GO analysis did not reveal statistical differences in the enrichment of GO slim terms using agriGO (Fisher’s exact test and the Yekutieli (false-discovery rate under dependency) multi-test adjustment method) [39]. However, this can partially be attributed to the incomplete annotation of the sorghum genome, as well as stage and tissue-specific expression not captured in our sample collection.

**Figure 7:**
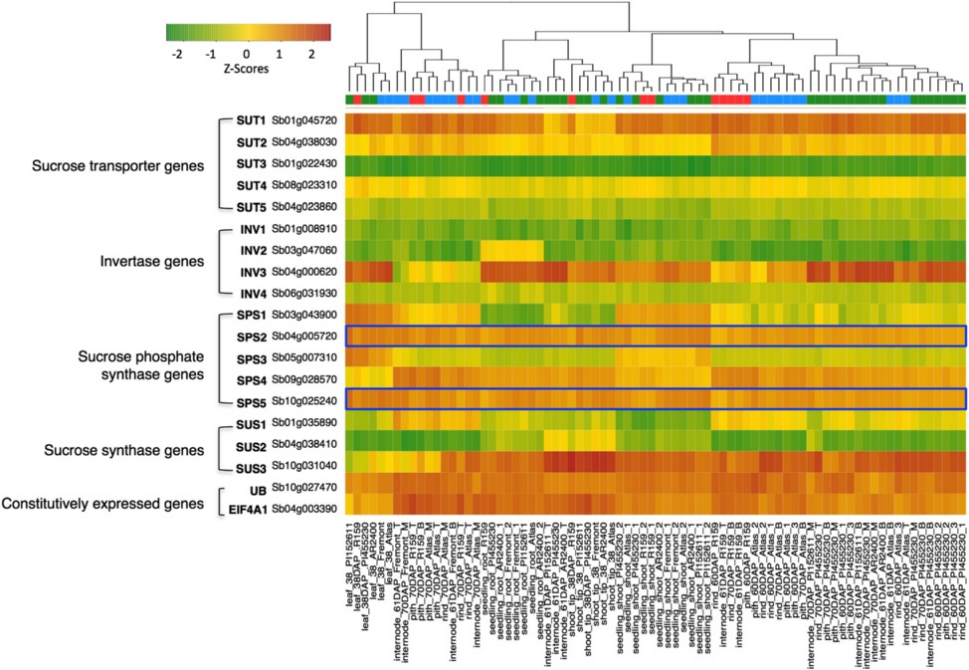
**Hierarchical clustering of samples based on expression of sucrose metabolizing enzymes and sucrose transporter genes.** Color bar key: Blue: sweet sorghum; red: grain sorghum; green: high biomass sorghum. Outlined in blue, the expression of sucrose phosphate synthase genes, SPS2 and SPS5, is consistently lower in grain types sweet and high biomass lines. Sugar metabolism gene list is appropriated from current literature [40].

To identify genotype-specific expression patterns, we examined the expression of several known sugar metabolizing enzymes and sucrose transporters in sorghum with the hypothesis that differential expression of these genes would be observable across genotypes (Additional file 12). Differential expression between sweet and grain sorghum has recently been shown [21,40], and our results further validate this observation, with the majority of sugar-related genes showing differential expression among tissues and genotypes. For example, sweet and high biomass varieties showed consistently higher expression of SPS2 and SPS5, sugar phosphate enzymes thought to play significant roles in sucrose biosynthesis, compared to grain varieties (Figure 7). A comprehensive gene list and more detailed expression analysis of sugar related genes across genotypes may provide insight into the mechanisms governing trade-offs in sorghum grain yield and stem sugar content.

**Figure 8:**
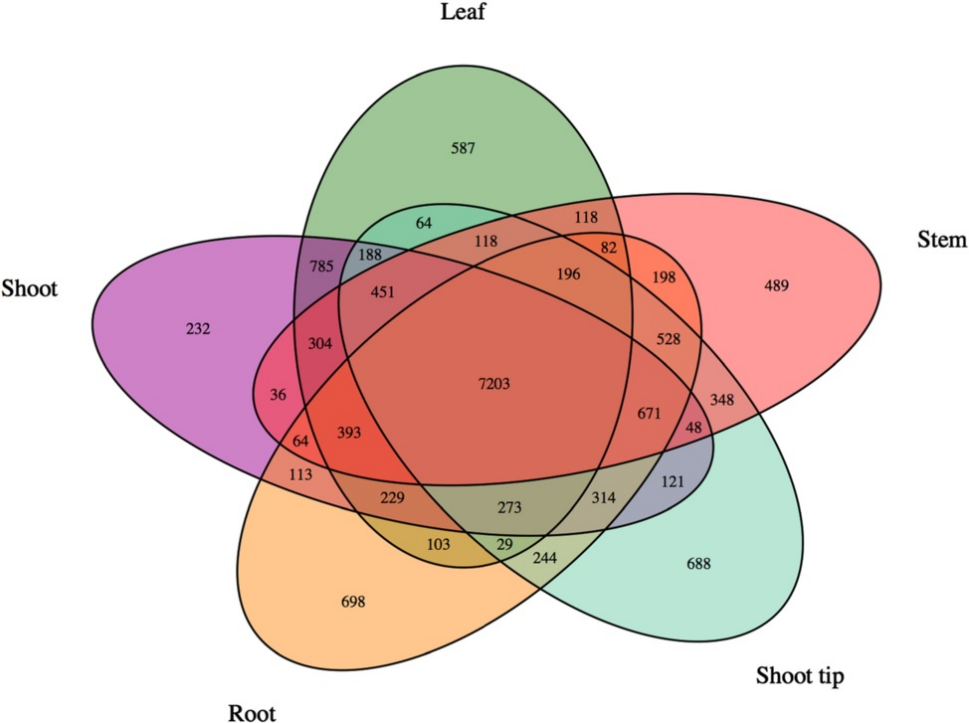
Number of shared and specific expression profiles of genes expressed in multiple tissue types (Atlas genotype).

**Figure 9:**
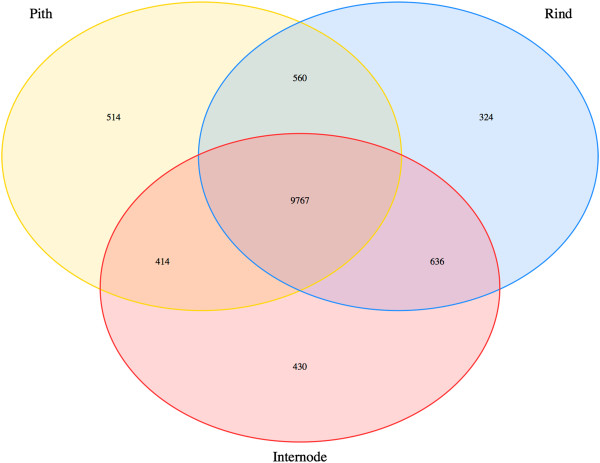
Number of shared and specific expression profiles of genes expressed in multiple stem tissues (Atlas genotype).

We further analyzed tissue-specific transcripts to identify shared and specifically expressed genes in multiple tissues (Figures 8 and 9). To avoid variation in gene expression due to genotypic differences, we chose samples from the genotype Atlas for this analysis. We identified 587, 489, and 698 genes that are specifically expressed in leaf, stem and root and 232 and 688 unique genes that are expressed in shoot and shoot tips, respectively (Figure 8). We also identified 960 genes that are specifically expressed in stem rind (predominantly lignified sclerenchymatous cells) as compared to 928 genes that are specifically expressed in stem pith (predominantly non-lignified parenchymatous cells; Figure 9). This dataset provides a unique opportunity to discover target sets of genes in core sorghum varieties that may be useful for modulating gene expression in a tissue-dependent manner. For example, these rind and pith-specific genes can be studied as potential candidate genes for biomass content and targets for compositional modification of biofuel feedstocks. Further, identification of promoter elements and corresponding DNA-binding regulatory proteins that regulate tissue-specific expression of genes could be identified from these data. As a direct application of this study, we are currently analyzing the promoter regions of candidate genes that are differentially expressed in the rind versus pith region of stem tissues.

### Tissue-specific expression of genes involved in the phenylpropanoid-monolignol pathway

To exemplify the functional utility of this data, we highlighted the expression data of 10 key enzymes associated with the phenylpropanoid-monolignol biosynthesis pathway (Additional file 13). Currently, one of the primary strategies for bioenergy feedstock improvement is through lignin modification. Alterations in lignin content and composition aim to improve the digestibility of forage and saccharification efficiency of lignocellulosic biofuels [41,42]. Thus, modifying the expression of genes in the lignin biosynthesis pathway is an attractive approach to achieving this goal.

**Figure 10:**
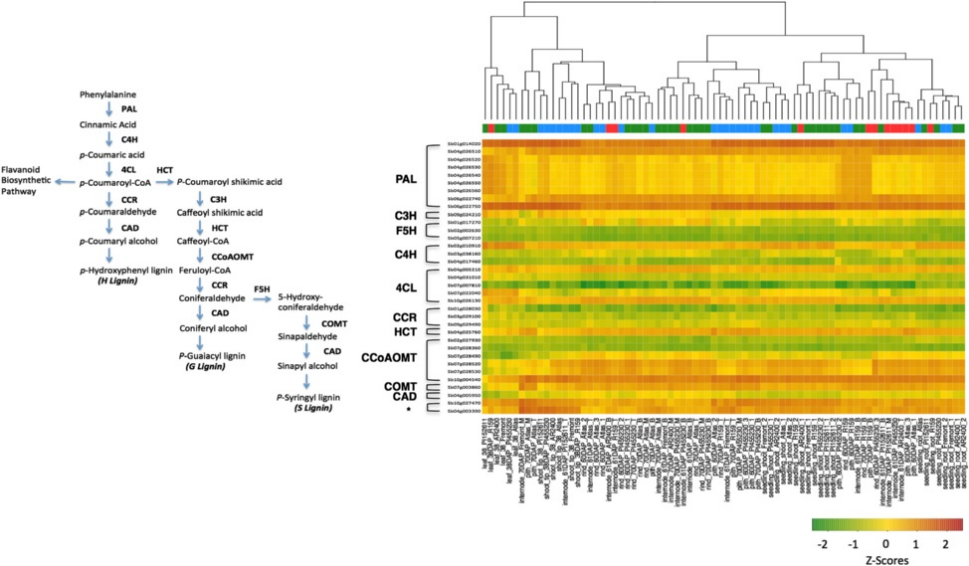
**Hierarchical clustering of tissues based on expression of phenylpropanoid-monolignol biosynthesis pathway genes.** *Constitutively expressed genes: Ubiquitin: Sb10g027470; EIF4A1: Sb04g003390. Color bar key: Blue: sweet sorghum; red: grain sorghum; green: high biomass sorghum. The color scale indicates the relative gene expression (Z-scores). Red, yellow, and green represent high, medium, and low levels of gene expression, respectively. The phenylpropanoid-monolignol pathway and enzyme nomenclature is appropriated from current literature [44].

Annotated in several databases, the majority of known and putative genes and homologs were analyzed for: phenylalanine ammonia-lyase (PAL, 9 sequences), coumaroyl shikimate 3’-hydroxylase (C3’H, 1), ferulate 5-hydroxylase (F5H, 3), cinnamate 4-hydroxylase (C4H, 3), 4-coumarate:CoA ligase (4CL, 5), cinnamoyl CoA reductases (CCR, 3), hydroxycinnamoyl CoA:shikimate hydroxycinnamoyl transferase (HCT, 1), caffeoyl-CoA 3-O-methyltransferase (CCoAMOT, 6), caffeic acid 3-O-methyltransferase (COMT, 1), and cinnamyl alcohol dehydrogenase (CAD, 1). Similar to previous studies in maize and switchgrass, the highest expression of these genes was found in the roots and stems [8,43]. Further, hierarchical clustering reveals that the expression of lignin biosynthesis genes varies with developmental stage, as well tissue type and genotype (Figure 10). Distinct expression signatures of gene homologs as well as clustering of above-ground vegetative tissues according to developmental stage has precedence in maize and, in general, most of the lignin genes showed organ-specific expression patterns consistent with studies in related species [24,38].

## Conclusions

Comprehensive transcriptome profiling provides a global overview of gene networks and allows for the discovery of functional connections between genes, mRNAs and their regulatory proteins. In the present study, we constructed a gene expression atlas covering an array of tissues, developmental stages and genotypes using the first commercially available sorghum microarray (Sorgh-WTa520972F). We observed tissue and genotype-specific expression patterns of relevant metabolic pathways that highlight the significance of intra-species variation in sorghum.

Developed as a new resource for crop breeding and genomic discovery, Sorgh-WTa520972F is produced by Affymetrix and is available to the public research community. We are currently utilizing this microarray to identify differential gene expression related to key metabolic processes (*e.g.,* starch/lignin biosynthesis) for the identification of regulatory regions. Additional avenues for future study with this array are wide-ranging and can include gene expression profiling during abiotic/biotic stress, plant infection and disease establishment to investigate genetic mechanisms and applications to plant breeding and crop improvement. Detailed expression analysis of small RNAs included in the array design may also reveal key insights in diverse biological processes, including RNA-guided gene regulation. Sorgh-WTa520972F can also be utilized in quantitative trait locus (QTL) mapping and validation methods (*e.g.,* identify differentially expressed genes from 'tolerant’ versus 'sensitive’ varieties). Minimal costs associated with microarray analysis allow for the generation of high-throughput expression profiles or combinations of profiles of elite breeding lines for accelerated crop-breeding efforts. Applications of this resource can target numerous agronomic traits in sorghum as well as provide insight in closely related grasses (*e.g.,* sugarcane, switchgrass, *Miscanthus x giganteus*) for improved feedstock development.

## Methods

### Tissue collection

To study the sorghum transcriptome and build the present gene expression atlas, we collected 78 samples from various developmental stages and tissue types (Additional file 1). Six diverse sorghum genotypes were grown in Chromatin’s greenhouse and field sites (Champaign, IL). These six genotypes were chosen to represent ideotypes of sorghum cultivation, including sweet, grain and high biomass sorghum varieties. Greenhouse grown seedling shoot and root samples were collected at 10DAP, which is roughly five days after plant emergence. Whole leaf and meristematic shoot tip samples were collected at 38DAP. This time-point captures the active growth phase of vegetative structures, including leaves, shoots and tillers. The stem tissue samples were collected at two time points: 61 and 70DAP. At 61DAP, the stem is fully formed in both flowering and non-flowering types. In flowering types, the head is also fully formed, and the period between 61 and 70DAP is a stage of active metabolism, capturing the transition between flowering (61DAP) and active grain filling (70DAP) [45]. The stem tissue was further dissected into the pith and the rind. As a bioenergy crop, the majority of fermentable sugar available in sorghum is present in the pith. The majority of lignin, however, is found in the rind [46]. Two tissue types (shoot and root) were represented by two biological replicates.

### Microarray design

A whole-transcriptome exon array for *Sorghum bicolor*: *Sorgh-WTa520972F* was designed and utilized for the present expression study. The array contains 1,026,373 probes covering 149,182 exons (27,577 genes) across the *Sorghum bicolor* nuclear, chloroplast and mitochondrial genome. The sequences used to construct the probesets included all identified *Sorghum bicolor* exons from the Sbi1 assembly and Sbi1.4 annotation (http://phytozome.net). We also added sequences for putative non-coding RNAs in *Sorghum bicolor* that may play a role in gene regulation (*e.g.,* rRNAs, tRNAs, snoRNAs and microRNAs). Confirmed functional small RNAs in closely related species (maize, sugarcane) were also included in our array design (http://bioinformatics.cau.edu.cn/PMRD, http://www.ncrna.org/frnadb) (Additional file 10).

### RNA Isolation and hybridization

Total RNA from all tissue types was extracted using a NucleoSpin RNA Plant Kit (Maxherey-Nagel, Germany). RNA integrity, as indicated by the detection of discrete ribosomal subunits, was verified electrophoretically. The RNA quality and quantity was further validated with a NanoDrop spectrophotometer (NanoDrop Technologies, Wilmington, DE). Prior to hybridization, the total RNA profile was also analyzed with Agilent 2100 Bioanalyzer (Agilent technologies, Waldbronn, Germany). Synthesis of cDNA, probe labeling and hybridization was performed by Precision Biomarker (Precision Biomarker Resources, Inc. Evanston, Illinois)

### Data extraction and evaluation of gene expression

Background correction and normalization were performed using a robust multi-chip average (RMA) algorithm in the Bioconductor *Affy* package [13]. Present calls for expressed genes were determined following established methods [24]. In brief, an expressed gene was identified by a RMA-normalized linear expression of >/= 320 in at least one of the 78 samples. The expression cut-off was five times the mean RMA-normalized signal from 576 negative-control oligos selected from the intronic regions of known constitutive genes (*e.g.,* actin, ubiquitin, and eIF4a1). A mean signal intensity of 64 was determined for the negative control oligos analyzed across all 78 slides. Constitutively expressed genes were identified by a RMA-normalized linear expression value of >/= 320 in all 78 samples.

### Principal component analysis, hierarchical clustering and z-scores

To study the biological relatedness and identify expression trends among the samples, we utilized the cmdscale function and then plotted using R. We used RMA-normalized log_2_ normalized expression values in the PCA analysis. Hierarchical clustering was performed using RMA-normalized log_2_ normalized expression values and clustered using Pearson’s correlation analysis. The Z scores were calculated as follows: Z = (X-X_mean_)/SD, where X is the average expression of a given gene in a tissue, and X_mean_ and SD are the mean expression and standard deviation respectively of that gene across all the selected tissues.

### GO Slim enrichment analysis

We evaluated enrichment of GO slim terms of biological process category (http://geneontology.org/GO.slims) in agriGO (http://bioinfo.cau.edu.cn/agriGO/) by Fisher’s exact test (p-value ≤0.05) and the Yekutieli (false-discovery rate under dependency) multi-test adjustment method [39].

### qRT-PCR

The relative mRNA expression was measured using Peltier Thermal Cycler PTC-200 PCR machine (MJ Research, Waltham, MA, USA) and the SuperScript III Platinum SYBR Green One-Step qRT- PCR kit (Invitrogen, Carlsbad, CA). Three independent reverse transcription reactions were performed for each RNA sample, and qRT-PCR was carried out under the following conditions: 100 nanograms of each RNA sample was reverse transcribed at 60°C for 3 minutes, and reverse transcription was followed by initial activation at 95°C for 5 minutes, and 40 amplification cycles at 95°C for 15 s and 50°C for 30s. Results were analysed using MJ Opticon Monitor 3.1.32 software, and relative expression of mRNA was calculated by the comparative Ct method (2^-[Δ][Δ]Ct^) [47]. Gene expression values across tissue types were normalized to ubiquitin expression.

### Availability of supporting data

The transcriptome dataset supporting the results of this article is available through NCBI's Gene Expression Omnibus (GEO) under accession number GSE49879, and the Sorghum Genome Array is available through Affymetrix (http://affymetrix.com).

## Competing interests

The authors declare that they have no competing interests.

## Authors’ contributions

NS wrote the manuscript, contributed for microarray design, carried out RNA isolations, validation assays, data analysis and interpretation of the results. RN and OC contributed to experimental and microarray design and participated in tissue sampling and phenotyping. GM and AF participated in data analysis. SK participated in analysis and interpretation of the results. All authors read, revised and approved the final manuscript.

## Supplementary Material

Additional file 1 Sorghum samples included in the gene expression atlas. Click here for file

Additional file 2 Phenotypic characteristics of sorghum genotypes included in gene expression atlas. Click here for file

Additional file 3 **Correlation of RNA expression between Illumina RNA sequencing and Affymetrix GeneChip microarray platform.** Each point represents a sorghum gene identified in grain sorghum leaf tissue of BTx623 by RNA-Seq, and by microarray in R159. RNA-Seq expression levels were measured using RPKM [19] and array levels were measured using the mean intensity of sense probes within exons. The Spearman’s coefficient is 0.61, which is consistent with previous studies and indicates that the platforms correlate well on similar samples. Click here for file

Additional file 4 Pearson's correlation coefficient of the biological replicates. Click here for file

Additional file 5 A: Expression dynamics across multiple tissue types detected by microarray and qRT-PCR. B: Pearson's correlation between expression levels determined by microarray and qRT-PCR. Click here for file

Additional file 6 Expression of sorghum homologs with established patterns of expression in related species. Click here for file

Additional file 7 **Number of genes expressed in each of the 78 samples.** Total: number of gene expressed in at least one organ (19,354; 70% of all genes on the array). Common: genes expressed in all 78 tissue types (4526; 15% of all genes on the array). Click here for file

Additional file 8 Gene Ontology classifications in the biological processes category identified using the AgriGO Singular Enrichment Analysis (SEA). Click here for file

Additional file 9 Identification of stably expressed genes. Click here for file

Additional file 10 List of small RNAs (sorghum, maize and sugarcane) included in microarray design. Click here for file

Additional file 11 **Number of tissue-specific small RNAs across sorghum ideotypes.** AR2400: biomass sorghum; Fremont: sweet sorghum; PI152611: forage sorghum; Common: number of genes in common among all three ideotypes. Click here for file

Additional file 12 Expression of select sucrose metabolizing enzyme/transporter genes. Click here for file

Additional file 13 Expression of select phenylpropanoid-monolignol biosynthesis pathway genes. Click here for file
