# Supplementary figures and images for: A Sorghum bicolor expression atlas reveals dynamic genotype-specific expression profiles for vegetative tissues of grain, sweet and bioenergy sorghums
Source: BMC Plant Biol. 2014 Jan 23;14:35. doi: 10.1186/1471-2229-14-35 (PMC3925131; doi:10.1186/1471-2229-14-35)

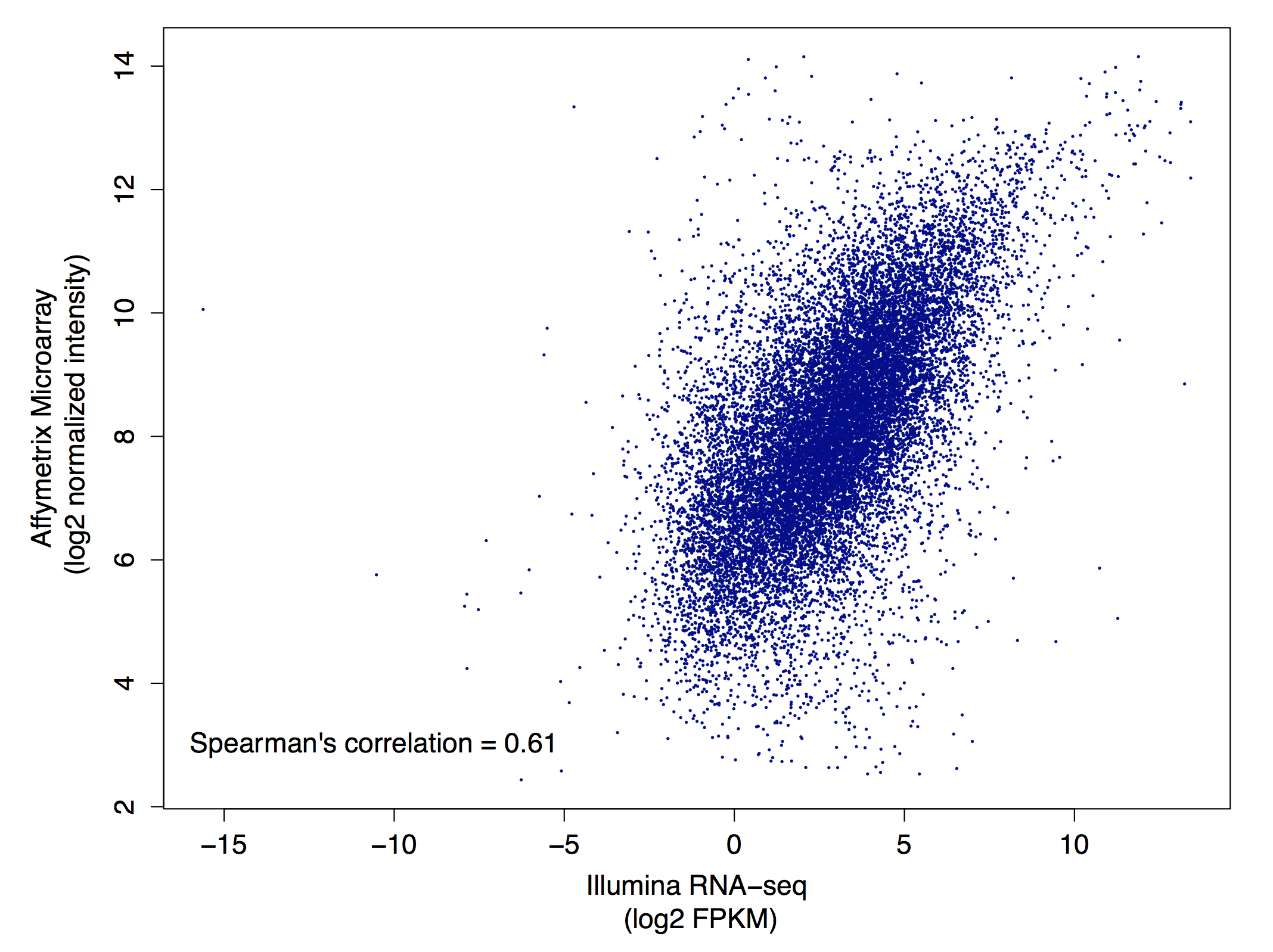

Supplement: Additional file 3 — Correlation of RNA expression between Illumina RNA sequencing and Affymetrix GeneChip microarray platform. Each point represents a sorghum gene identified in grain sorghum leaf tissue of BTx623 by RNA-Seq, and by microarray in R159. RNA-Seq expression levels were measured using RPKM [19] and array levels were measured using the mean intensity of sense probes within exons. The Spearman’s coefficient is 0.61, which is consistent with previous studies and indicates that the platforms correlate well on similar samples. [file 1471-2229-14-35-S3.png]

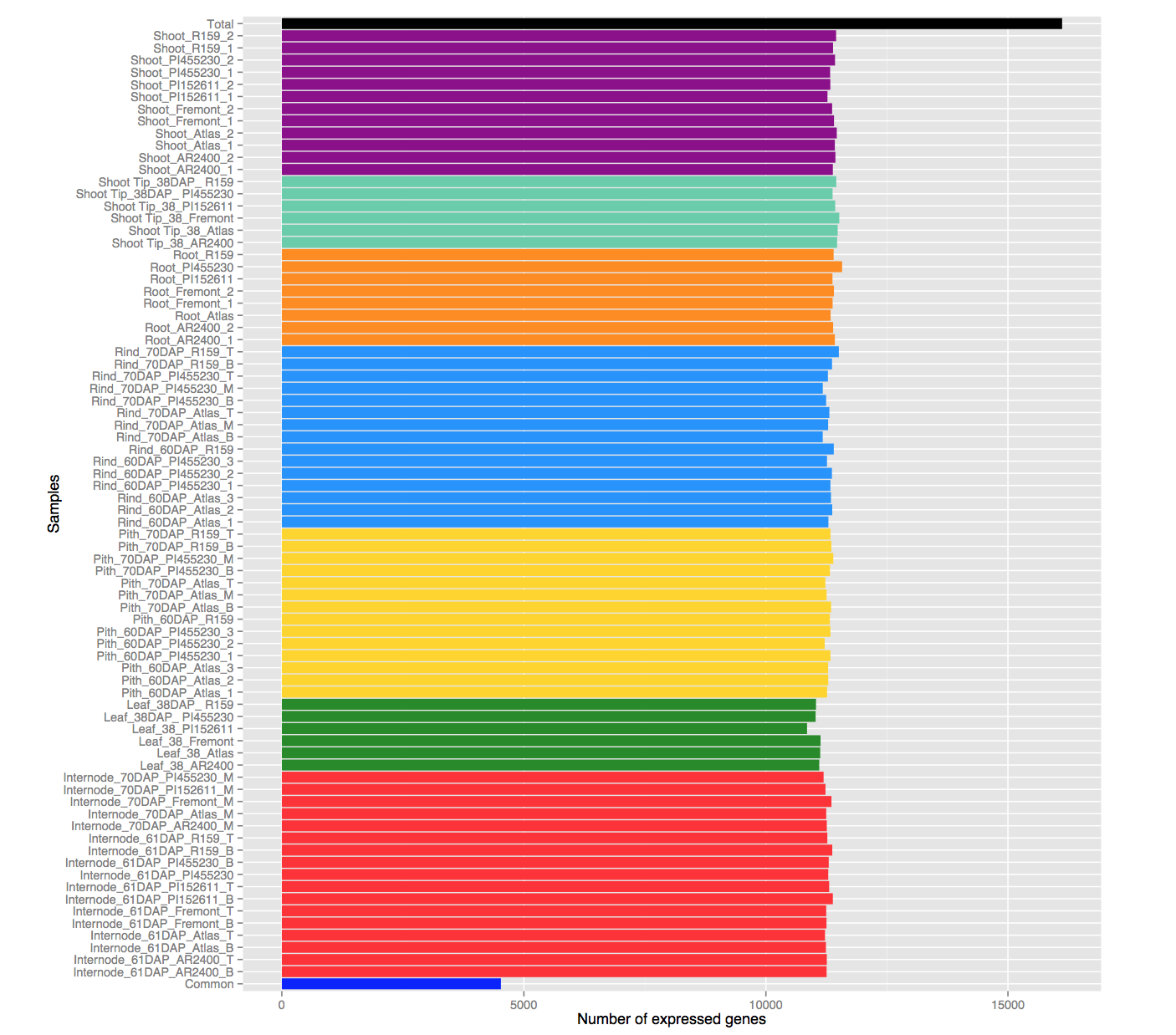

Supplement: Additional file 7 — Number of genes expressed in each of the 78 samples. Total: number of gene expressed in at least one organ (19,354; 70% of all genes on the array). Common: genes expressed in all 78 tissue types (4526; 15% of all genes on the array). [file 1471-2229-14-35-S7.png]

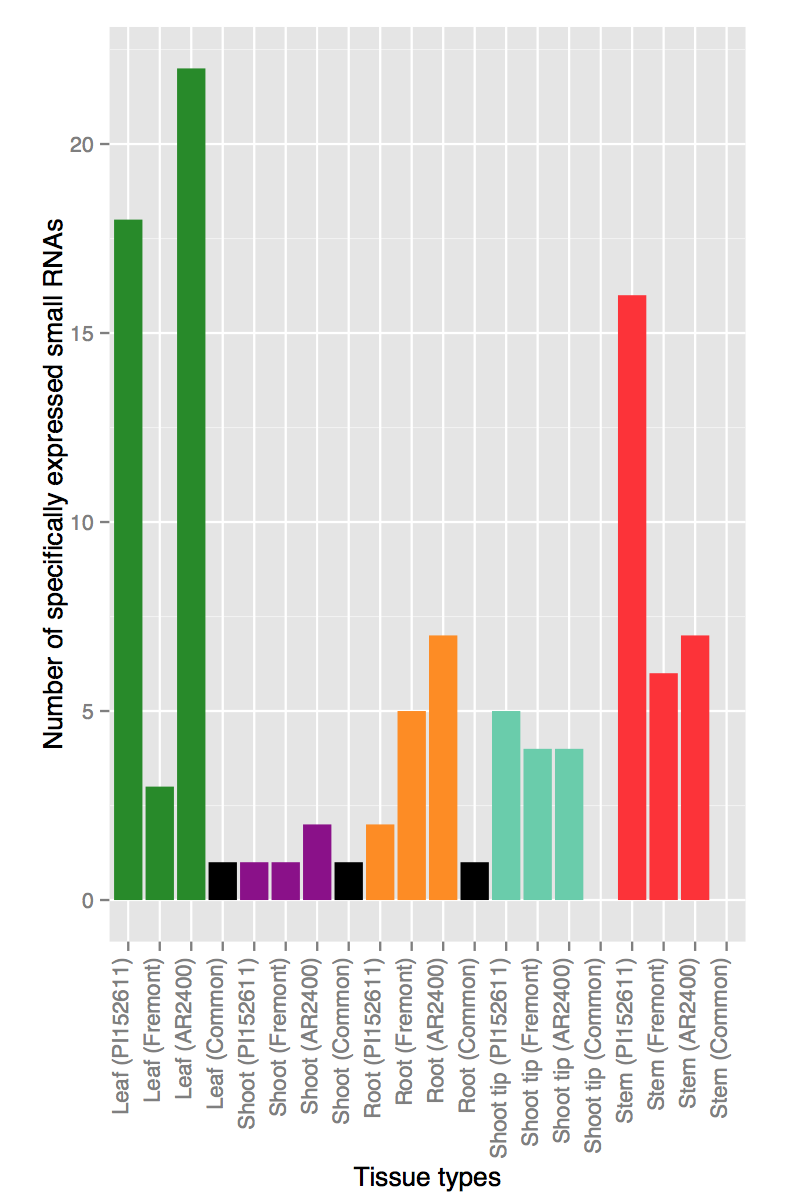

Supplement: Additional file 11 — Number of tissue-specific small RNAs across sorghum ideotypes. AR2400: biomass sorghum; Fremont: sweet sorghum; PI152611: forage sorghum; Common: number of genes in common among all three ideotypes. [file 1471-2229-14-35-S11.png]
